# An Oversampling-Enhanced Multi-Class Imbalanced Classification Framework for Patient Health Status Prediction Using Patient-Reported Outcomes

**DOI:** 10.1109/access.2025.3617316

**Published:** 2025-10-03

**Authors:** YANG YAN, ZHONG CHEN, CAI XU, XINGLEI SHEN, JAY SHIAO, JOHN EINCK, RONALD C. CHEN, HAO GAO

**Affiliations:** 1 School of Computing, Southern Illinois University, Carbondale, IL 62901, USA; 2 Heritage College of Osteopathic Medicine, Ohio University, Athens, OH 45701, USA; 3 Department of Radiation Oncology, The University of Kansas Medical Center, Kansas, KS 66160, USA; 4 Department of Radiation Oncology, UT Southwestern Medical Center, Dallas, TX 75390, USA

**Keywords:** Patient-reported outcomes, multi-class classification, oversampling, skewed class distribution, radiation therapy

## Abstract

Patient-reported outcomes (PROs), directly captured from cancer patients undergoing radiation therapy, play a crucial role in guiding clinicians’ counseling on treatment-related toxicities. Accurate prediction and assessment of symptoms and health status linked to PROs are essential for improving clinical decision-making and planning post-treatment support as patients transition into survivorship. However, raw PRO data collected in clinical settings presents two inherent challenges, including data sparsity (due to incomplete item responses) and imbalanced toxicity distributions. These factors complicate predictive modeling. This study investigates advanced machine learning techniques to address these challenges by predicting outcomes such as pain and sleep disturbances using PRO datasets from a cancer therapy center. We implement advanced classifiers (i.e., RF, XGBoost, GB, SVM, MLP-Bagging, and LR) for multi-class imbalance tasks across three cancers. To address the minority cases, we apply oversampling while preserving class ratios. Experimental results demonstrate RF and XGBoost’s strong generalization, highlighting their utility in categorizing post-therapy severity levels for clinical decision support.

## I. INTRODUCTION

Patient-reported outcomes (PROs), including health-related quality of life, pain severity, sleep disturbances, and depressive symptoms, are widely used in observational studies and clinical trials to evaluate patient health and treatment impacts at the population level [1], [2]. These outcomes hold enduring significance in global clinical and academic research, driving the creation of numerous validated instruments to systematically quantify patients’ self-assessed health status. However, despite the proliferation of PRO tools, their clinometric properties, such as reliability, validity, and responsiveness, remain understudied [3]. Artificial intelligence (AI) and machine learning (ML) offer advanced data modeling and predictive capabilities that are increasingly recognized as transformative tools for addressing this gap. AI/ML methodologies allow researchers to rigorously evaluate and enhance the psychometric robustness of PRO measures [4]. This ultimately improves their accuracy and clinical usefulness in both research and patient care.

**FIGURE 1.**
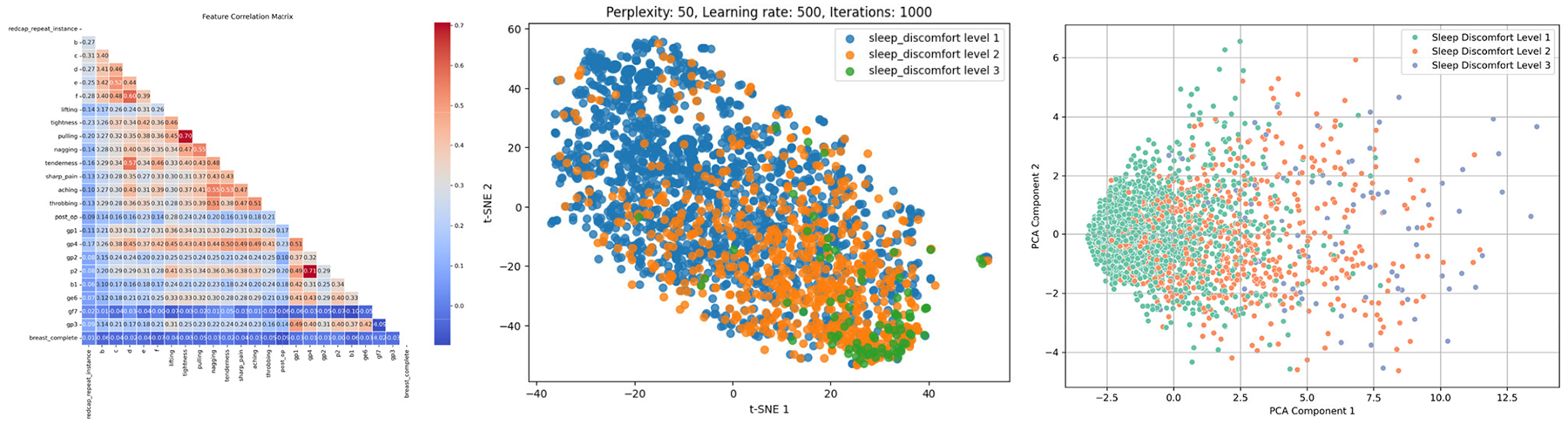
The right panel shows the feature correlation matrix for breast cancer patients’ PRO. Multi-dimensional reduction and visualization with t-SNE (middle panel) and PCA (left panel) for breast cancer patients with sleeping discomfort levels 1–3. Each point represents a breast cancer patient, and each color represents the corresponding health status such as sleep discomfort level.

While substantial psychological research has focused on the period of cancer diagnosis and long-term survivorship, the psychological consequences of treatment cessation, particularly following radiation therapy, remain understudied. Longitudinal investigations [5], [6], [7], [8] reveal a counter-intuitive trend: anxiety and depressive symptoms frequently escalate in patients after therapy concludes. Several factors may cause this phenomenon. Among them are the abrupt end of structured medical support and the loss of treatment as a psychological anchor. Diminished social reinforcement and persistent fears of recurrence also contribute. PROs are critical for capturing patient experiences across the continuum of radiation therapy. Clinically, stratifying patients by outcome severity, particularly identifying those with severe symptoms, is essential for targeted care. However, two key challenges hinder this process. First, PRO datasets often exhibit pronounced class imbalance: disproportionately fewer patients report severe pain, depression, or discomfort compared to mild or moderate cases, despite the former group’s need for heightened clinical attention. Second, patient heterogeneity, as illustrated in Figure 1 via t-SNE (middle panel) and principal component analysis (PCA) (left panel) visualization [25], complicates analysis. Both t-SNE (a non-linear method preserving local structure) and PCA (a linear technique maximizing global variance) reduce the dimensionality of the complex PRO data (right panel in Figure 1). However, they consistently fail to reveal distinct clustering among the three predefined subgroups of breast cancer patients (based on sleeping discomfort levels 1–3). The substantial overlap observed between these subgroups in both visualizations underscores this key challenge. Such heterogeneity manifests in clinical parameters, demographics, molecular profiles, treatment responses, and tumor subtypes, mirroring the broader biological complexity of intra-tumor heterogeneity observed across malignancies.

**FIGURE 2.**
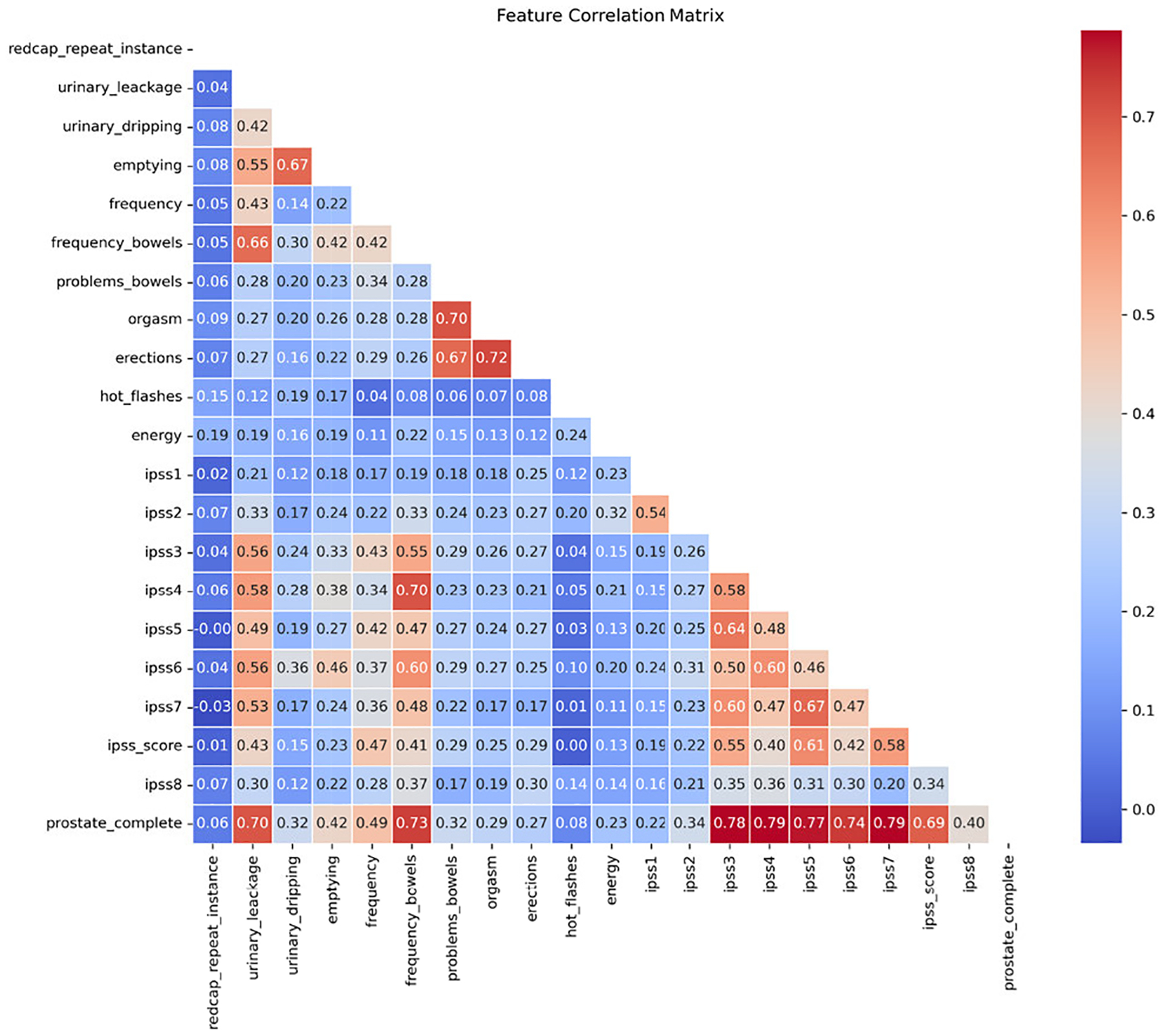
Feature correlation matrix for prostate cancer patients’ PRO.

Figure 2 presents the feature correlation matrix for prostate cancer patients’ PRO data, using a color gradient to visualize correlation strength: red indicates strong positive correlations, light blue mild positive correlations, and blue weak correlations. A strong positive correlation (*r* > 0.70) was observed between ‘prostate completeness’ and several features, including urinary leakage, bowel frequency, and multiple IPSS (International Prostate Symptom Score) items (e.g., ipss3–7). Sexual health features also show strong interdependence, particularly between orgasm and erection (*r* = 0.72). Conversely, several weak negative correlations highlight feature independence. Notably, ‘energy map’ exhibits only mild correlations with most other features (*r* = 0.12 – 0.24), suggesting its variation is largely independent of urinary or sexual symptoms; a similar pattern is observed for ‘hot flashes’ and ‘ipss1’. The ‘redcap_repeat_instance’ variable, reflecting REDCap’s longitudinal data collection functionality, demonstrates weak correlations with all other PRO features, consistent with its role as an administrative indicator rather than a clinical symptom measure.

The assumption of uniform misclassification costs across classes in PRO datasets is invalid. For example, in predicting severe outcomes, failing to identify a severe case (false negative) is clinically riskier than misclassifying a mild case as severe (false positive). Multi-class imbalance classification [9], [34], [35] introduces critical challenges that undermine model performance, particularly in high-stakes domains like PRO analysis: (1) Model bias toward majority classes [36], [37]: Algorithms trained on imbalanced data frequently exhibit bias toward majority classes. Conventional learning paradigms prioritize overall accuracy, inadvertently neglecting minority class patterns. (2) Overfitting to majority classes [38], [39], [40]: Repeated exposure to majority class examples increases the risk of overfitting to spurious correlations or dataset artifacts, reducing generalizability to underrepresented (but clinically critical) minority classes. (3) Diminished sensitivity for minority classes [33], [41], [42]: Minority classes suffer from inadequate representation, leading to poor sensitivity (true positive rate). In clinical contexts, this poses significant risks, as failing to detect rare but severe outcomes such as acute pain or depression, can delay interventions. These challenges are exacerbated in PRO datasets, where severe outcomes are inherently sparse yet demand prioritized attention. Addressing these issues is essential for developing models that align with clinical priorities and improve patient care.

Rapid progress in AI and ML has spurred the development of advanced algorithms to tackle multi-class imbalanced classification challenges [9], [34], [35]. Current strategies to mitigate class imbalance broadly fall into three categories: data-level methods, algorithm-level adaptations, and ensemble learning techniques. Each approach provides unique mechanisms to address issues such as model bias, overfitting, and reduced sensitivity, thereby improving the robustness and reliability of predictive models in high-stakes applications like clinical decision-making.

Data-level methods mitigate class imbalance by resampling datasets to modify class distributions. These techniques [58] either augment minority classes (e.g., oversampling) or reduce majority class representation (e.g., undersampling). While oversampling synthesizes new minority-class instances through interpolation, undersampling discards majority-class data to prevent model bias. SMOTE, for instance, generates synthetic samples by interpolating feature-space neighbors, improving minority-class generalization. However, oversampling risks overfitting to noise, while over-aggressive undersampling may discard informative majority-class patterns, degrading model performance.

Algorithm-level methods adapt learning procedures to counteract imbalance-induced bias [22]. These approaches modify components of the training process that induce majority-class bias, such as adjusting loss functions or decision thresholds. A prominent strategy is cost-sensitive learning [29], [30], [31], [32], [33], which assigns higher misclassification penalties to minority classes. By prioritizing rare but critical errors (e.g., false negatives in severe outcome prediction), cost-sensitive frameworks shift decision boundaries to improve minority-class sensitivity. However, their efficacy depends on accurate cost assignment and classifier compatibility, necessitating domain-specific tuning.

Ensemble methods [26], [27], [28], [59] combine multiple base classifiers to enhance robustness against imbalance. Techniques like boosting iteratively train classifiers on reweighted data, focusing on misclassified minority instances in subsequent iterations. For example, AdaBoost increases weights for hard-to-classify samples, forcing later classifiers to prioritize them. This iterative refinement reduces both bias and variance, improving generalization across classes. Similarly, bagging generates diverse subsets via bootstrap sampling, aggregating predictions to stabilize performance. By synergizing resampling with model aggregation, ensembles address imbalance while mitigating overfitting, making them particularly effective for complex, heterogeneous PRO datasets.

Accurate assessment of PROs often necessitates comprehensive data collection beyond standard clinical metrics. However, patient concerns regarding data security and privacy may lead to incomplete PRO submissions, compounding the challenge of deriving meaningful insights from sparse or fragmented datasets. To address these issues, this study investigates machine learning classifiers specifically tailored for multi-class imbalanced PRO data, aiming to ensure robust and equitable symptom evaluation. Our methodology employs a three-stage preprocessing pipeline: (1) Iterative imputation: Leverages conditional distribution learning to address missing data while preserving dataset structure. (2) Normalization: Applies label encoding and standard scaling to harmonize heterogeneous feature ranges. and (3) Strategic oversampling: Adjusts class representation via interpolation-based techniques that maintain the original skewed distribution, ensuring minority classes are amplified without distorting inherent data patterns. We evaluated six established classifiers across prostate, head and neck, and breast cancer cohorts. Experimental results demonstrate that RF and XGBoost consistently outperform baseline models, achieving superior classification accuracy and clinically interpretable feature importance rankings. These findings underscore their potential for real-world deployment, where balancing data constraints with actionable clinical insights is critical.

The main contributions of our research to the field of PRO can be summarized as follows.

1. We provide a comprehensive evaluation of a set of classifiers, including Support Vector Machine (SVM), Logistic Regression (LR), Gradient Boosting (GB), XGBoost (XGB), Random Forest (RF), and Bagging with Multi-Layer Perceptron (Bagging-MLP), alongside an oversampling strategy to tackle multi-class imbalance in PRO datasets.
2. We detail the feature interpretations related to PRO, derived from questionnaires administered before, during, and after radiation therapy.
3. We report on the classification performance and time efficiency of top ML classifiers, offering insights that guide their clinical use.

The rest of the paper is organized as follows. The related work is discussed in Section II. The utilized approaches are elaborated in Sections III. The experimental results are reported in Section IV. The conclusion is summarized in Section V.

## II. RELATED WORK

In this section, we review the existing exploratory studies on PRO and state-of-the-art ML for PRO prediction.

### A. EXISTING EXPLORATORY STUDIES ON PRO WITH RADIATION THERAPY

PROs [43], [45] have become increasingly prominent in radiation therapy research due to their capacity to quantify patients’ subjective experiences during treatment, enabling clinicians to assess toxicity profiles across chemotherapy, immunotherapy, and radiotherapy regimens. For instance, Grover et al. [46] conducted a comparative analysis of patient-reported toxicities in prostate cancer treated with multimodal therapies sharing similar oncological endpoints. Their findings revealed that men undergoing prostate cancer treatment experienced substantial urinary and sexual side effects across all modalities, with surgical interventions associated with higher rates of such complications compared to radiation-based approaches. Complementing this, Oh et al. [47] applied confirmatory factor analysis to PRO datasets, identifying clinically coherent symptom clusters and establishing correlations between these groupings and radiation dose parameters. These studies underscore PROs’ dual utility in both evaluating treatment-related morbidity and guiding dose optimization strategies.

Barocas et al. [48] assessed functional outcomes and treatment-related morbidity in 2,550 men undergoing radical prostatectomy, external beam radiation therapy (EBRT), or active surveillance for prostate cancer. Their prospective population-based cohort study revealed that radical prostatectomy correlated with significantly greater declines in sexual function at 3 years relative to EBRT and active surveillance.

In a complementary investigation, Arbab et al. [49] explored mental health trajectories among cancer patients receiving radiation therapy. Using latent class analysis, they identified four distinct psychosocial trajectories characterized by varying anxiety, depression, and well-being patterns. The cohort predominantly included patients diagnosed with respiratory cancers and moderate-to-severe comorbidities, with sociodemographic, clinical, and symptom-related variables, such as physical symptom burden and social support, emerging as key predictors of these trajectories. These findings highlight the interplay between clinical factors and psychosocial outcomes in oncology care.

### B. STATE-OF-THE-ART MACHINE LEARNING METHODS FOR PRO

ML is increasingly being integrated into the analysis of PROs to enhance predictive accuracy, personalize treatment strategies, and improve patient care in radiation therapy and other medical domains [60], [61], [62], [63], [64]. For instance, Staartjes et al. [11] developed deep neural network and logistic regression models to predict patient-reported outcome measures (PROMs). Their study focused on the minimum clinically important difference (MCID), defined as a 30% or greater improvement from baseline in numeric rating scales for pain and the Oswestry Disability Index for functional disability. Among 422 included patients, 337 (80%), 219 (52%), and 337 (80%) achieved clinically meaningful improvements in leg pain, back pain, and functional disability, respectively, at the 1-year follow-up. The deep learning models demonstrated strong predictive performance, achieving area-under-the-curve (AUC) values of 0.87, 0.90, and 0.84, alongside accuracies of 0.85, 0.87, and 0.75 for the three endpoints.

Tschuggnall et al. [50] applied modern ML techniques to a real-world dataset of 1,047 rehabilitation patients to develop models predicting treatment success at the onset of therapy. By integrating clinical data and PROMs from questionnaires, they computed patient-specific CROMs, such as knee range of motion, and used these indicators to train predictive models. Pfob et al. [51] developed and validated three ML algorithms, LR with elastic net penalty, XGBoost, and neural networks, to predict clinically significant differences in satisfaction with breast outcomes using the validated BREAST-Q questionnaire at a 2-year follow-up. In a separate study, Sidey et al. [52] collected PRO data from a U.S. academic cancer institution, where patients with recurrent ovarian cancer completed biopsychosocial PROMs every 90 days. The dataset was randomly split into training and testing subsets at a 2:1 ratio, with synthetic minority oversampling applied to address class imbalance in the training cohort. Six ML algorithms were trained on the data, and their predictions on the test set were aggregated through a voting ensemble to enhance robustness. Yang et al. [53] applied ML algorithms and statistical modeling to investigate the relationship between radiation therapy and post-treatment gastro-urinary function. Due to limited patient datasets, they employed image flipping and curvature-based interpolation to augment their dataset, enabling transfer learning. Using this augmented data, they trained a convolutional autoencoder to derive near-optimal weight initializations. A subsequent convolutional neural network (CNN) was then used to analyze associations between patient-reported quality-of-life (QoL) outcomes and radiation doses delivered to the bladder and rectum. The study further incorporated analysis of variance (ANOVA) and logistic regression to assess organ-specific radiation sensitivity and establish dose thresholds for distinct anatomical regions. Their results revealed no statistically significant link between bladder radiation doses and QoL scores.

## III. METHODOLOGY

### A. PRO DATA COLLECTION AND PRE-PROCESSING

Our analysis focused on the three most prevalent cancer types in the dataset: prostate, head and neck, and breast cancer, selected based on case frequency. Pre-processing involved removing rows and columns with missing values (null/NaN) to ensure data integrity. To address class imbalance and incomplete data, we implemented a multi-step approach, (i) iterative imputation is to Leverage conditional distribution learning to estimate missing values. (ii) label encoding and standard scaling are applied to homogenize the dataset and feature normalization. and (iii) augmented minority class samples are used to mitigate imbalance.

We evaluated six classifiers, Logistic Regression (LR) [12], Support Vector Machine (SVM) [15], Gradient Boosting (GB) [16], XGBoost (XGB) [16], Random Forest (RF) [17], and Bagging with Multi-Layer Perceptron (MLP-Bagging) [13], to address multi-class imbalance in PRO datasets. A grid search with cross-validation was employed to systematically explore hyperparameter spaces (e.g., regularization strengths, tree depths, learning rates) across all models. This process aimed to identify configurations maximizing classification accuracy while alleviating overfitting.

### B. LOGISTIC REGRESSION (LR)

LR [12] is a linear statistical method that models binary outcomes using a logistic (sigmoid) function. Its key strengths include inherent interpretability and the ability to estimate class membership probabilities, which is particularly valuable for prioritizing critical clinical outcomes such as severe pain, reduced quality of life, or depressive symptoms following radiation therapy. Unlike many models, LR imposes no assumption of normality for predictor variables. Independent variables are encoded binary (0 or 1) to denote the absence (0) or presence (1) of the target outcome, while the model’s output, bounded between 0 and 1, represents the likelihood of outcome occurrence. The LR is based on the logistic function *P*, determined as $P=\frac{1}{1+e^{-X}}$, where *P* denotes the probability related to a certain observation, and *X* could be defined as *X* = *β*_0_ + *β*_1_*x*_1_ + *β*_2_*x*_2_ + ⋯ + *β*_*n*_*x*_*n*_, where *β*_0_ denotes the intercept of the algorithm, *β*_*i*_ is the coefficient representing the independent variables contribution *x*_*i*_, and *n* denotes the number of conditioning factors. Given its transparency in coefficient interpretation and probabilistic output, we include LR as a benchmark for interpretability in multi-class classification tasks.

### C. MULTILAYER PERCEPTRON-BAGGING (MLP-BAGGING)

Multilayer Perceptron (MLP) [13] is a class of feed-forward artificial neural networks (ANNs) composed of interconnected layers of neurons. Each neuron applies a linear transformation (weighted sum of inputs) followed by a non-linear activation function, enabling the network to model complex relationships. Neurons are organized in sequential layers, input, hidden, and output, with full connectivity between successive layers, allowing hierarchical feature learning. This architecture enables MLPs to approximate non-linear functions by using hidden layers to progressively transform input data through nonlinear activations. MLP-Bagging [14] extends the standard MLP through bootstrap aggregating (bagging), an ensemble technique designed to improve model robustness and generalization. Bagging creates diversity by training multiple MLP instances on bootstrapped subsets of the training data. The final prediction aggregates output from all models, reducing prediction variance and mitigating overfitting, particularly advantageous in noisy or high-dimensional datasets where single MLPs may exhibit instability. By combining ensemble-driven reliability with the representational capacity of neural networks, MLP-Bagging excels in scenarios requiring both accuracy and generalization ability.

### D. SUPPORT VECTOR MACHINE (SVM)

Support Vector Machines (SVMs) [15] are a class of kernel-based machine learning models renowned for their theoretical guarantees in optimizing generalization performance. Initially designed for classification and regression tasks, SVMs excel by constructing hyperplanes that maximize the margin in a high-dimensional feature space. This margin maximization principle not only minimizes empirical risk (training errors) but also enhances generalization to unseen data by avoiding overfitting. Key strengths of SVMs include their ability to solve convex optimization problems, ensuring globally optimal solutions, and their discriminative power in separating complex data distributions via kernel functions (e.g., linear, polynomial, or radial basis functions). These attributes have solidified SVMs as a cornerstone method in data mining, pattern recognition, and machine learning. Compared to traditional supervised learning approaches, SVMs often achieve superior performance in high-dimensional or non-linearly separable datasets, even with modest data volumes.

### E. GRADIENT BOOSTING (GB)

Gradient Boosting (GB) [16] is a powerful machine learning technique that iteratively constructs an ensemble of decision trees to address regression and classification tasks. Unlike standalone decision trees, GB employs sequential additive modeling—each subsequent tree corrects residuals from the previous models. This iterative refinement enhances predictive accuracy while maintaining interpretability and retaining decision trees’ inherent advantages, such as handling heterogeneous data types and non-linear relationships. Key strengths of GB include (i) reduced overfitting through stage-wise tree construction and regularization, (ii) tolerance for missing data, outliers, and large datasets without extensive pre-processing, and (iii) effective handling of complex data structures via hierarchical feature interactions. By combining the simplicity of decision trees with ensemble-driven performance, GB outperforms traditional single-tree models in both stability and predictive power, making it a cornerstone method for modern data mining applications.

### F. XGBOOST

XGBoost [16] is an advanced gradient-boosting framework that builds ensembles of decision trees, optimized through a differentiable loss function. Unlike conventional ensemble methods, XGBoost iteratively refines predictions by sequentially training trees to correct errors from prior models, with final class probabilities derived via logistic transformations of aggregated tree outputs. The regularization, scalability, and flexibility innovations collectively enhance XGBoost’s robustness, computational efficiency, and adaptability to complex data patterns, solidifying its status as a state-of-the-art solution for high-dimensional, noisy, or heterogeneous machine learning problems.

### G. RANDOM FOREST (RF)

Random Forest (RF) [17] is an ensemble learning method that aggregates predictions from multiple decorrelated decision trees, each trained on a bootstrapped subset of the data and a random selection of features. Renowned for its classification accuracy and resistance to overfitting, RF operates by (i) growing individual trees through recursive binary splitting, starting from root nodes until stopping criteria are satisfied; (ii) combining predictions via majority voting (classification) or averaging (regression) across all trees. The method’s robustness stems from its ability to reduce variance by averaging high-bias, low-variance trees while minimizing inter-tree correlation through feature randomness. This ensures strong performance even in high-dimensional or noisy datasets.

### H. OVERSAMPLING

Oversampling techniques [18], [19], [20], [21] address class imbalance by augmenting minority classes through duplication or synthetic sample generation. While effective for rebalancing datasets, these methods can inflate computational costs due to increased training set size. Random oversampling duplicates existing minority samples but risks overfitting by reinforcing noise or outliers. To overcome these limitations, SMOTE [10] generates synthetic minority samples by interpolating between neighboring instances in the feature space (i.e., the multi-dimensional representation of attributes and their interactions). Unlike random duplication in the data space, SMOTE creates linearly combined examples within local neighborhoods, enriching the feature distribution while preserving underlying data structures. We applied SMOTE to synthesize minority class samples, enabling conventional ML algorithms to train on balanced datasets. The advantage of SMOTE over random oversampling lies in its ability to generate synthetic minority-class samples via linear interpolation, which mitigates overfitting risks compared to mere duplication as discussed in [10] and [55]. By integrating SMOTE into our pipeline, we leverage its proven efficacy in handling imbalanced data while maintaining methodological flexibility for diverse ML frameworks [44].

## IV. EXPERIMENTS

We conducted a series of experiments to verify the effectiveness of the ML approaches for the PRO data analysis.

### A. DATASETS

Table 1 presents the number of cases for each cancer type and the class distribution within the datasets. The head and neck dataset consists of 25 attributes and 2,525 training patients distributed across five classes. The notation (939:657:554:304:71) indicates the number of cases per class, with 939 patients in class 1, 657 patients in class 2, 554 patients in class 3, 304 patients in class 4, and 71 patients in class 5, respectively. After applying random oversampling to balance the class distribution, the training set increased to 4,695 patients, with equal representation across classes.

Similarly, the prostate dataset contains 22 attributes and 3,139 training patients spread across five classes, ranging from 1,477 patients in class 0 to 65 patients in class 4 (1,477:767:491:339:65). After oversampling, the training set expanded to 7,385 patients, ensuring equal representation for all classes. The test set consists of 784 patients, distributed as (362:208:123:79:12).

The breast dataset includes 24 attributes and 2,054 training patients distributed across three classes, with class 1 having 1,430 patients and class 3 having 69 patients (1,430:555:69). Following oversampling, each class contains 1,430 patients, totaling 4,290 training patients, while the test set has 514 patients distributed as (361:131:22).

### B. EVALUATION METRICS

We utilized precision, recall, F1-score, AUC score, ROC, confusion matrix, and average running time [23], [24] in multi-class imbalance classification as the evaluation metrics to measure the performance of all completing algorithms.

1. **Precision** measures the accuracy of positive predictions. It is the ratio of true positive (TP) predictions to the sum of true positive and false positive (FP) predictions. High precision indicates that the model has a low false positive rate.
$$\text{Precision}=\frac{TP}{TP+FP}$$
2. **Recall** measures the model’s ability to identify all relevant instances. It is the ratio of true positive (TP) predictions to the sum of true positives and false negatives (FN). High recall indicates that the model captures most of the positive instances.
$$\text{Recall}=\frac{TP}{TP+FN}$$
3. **F1-score** is the harmonic mean of precision and recall, providing a balance between the two metrics. It is particularly useful when dealing with imbalanced datasets, as it considers both false positives and false negatives.
$$F1-\text{score}=2\cdot \frac{\text{Precision}\cdot \text{Recall}}{\text{Precision}+\text{Recall}}$$
4. **AUC (Area Under the Curve)** represents the area under the ROC curve, providing a measure of the model’s ability to distinguish between classes. An AUC of 1 indicates perfect classification, while an AUC of 0.5 indicates no discrimination capability.
5. **ROC (Receiver Operating Characteristic) Curve** is a graphical representation of the true positive rate (sensitivity) against the false positive rate at various threshold settings. The ROC curve helps to visualize the trade-off between sensitivity and specificity.
6. **Confusion Matrix** is used to describe the performance of a classification model. It shows the true positives, true negatives, false positives, and false negatives, allowing for a detailed analysis of the model’s performance.

### C. IMPLEMENTATION DETAILS

For handling missing values in numerical columns, we employ the IterativeImputer method from the sklearn.impute library. Additionally, we use math.ceil() to round the imputed values to the nearest integer. The dataset is split into training and testing sets using five-fold cross-validation. For oversampling the training data, we apply the RandomOverSampler from the imblearn library. To optimize model performance, we employ GridSearchCV from sklearn.model_selection to fine-tune hyper-parameters for various ML algorithms, including SVM, LR, GB, XGB, and RF. The grid search will explore a range of hyper-parameters in each ML model, which is an exhaustive search of all the parameters in steps to obtain the best ones. The parameter settings of the ML models we selected are shown in Table 2.

### D. OVERALL COMPARISON

For three cancer datasets featuring diverse PRO outcomes, the comparative performance of all competing algorithms in terms of precision, recall, F1-score, and weighted AUC score is summarized in Table 3. Several observations regarding performance can be made. Firstly, Random Forest (RF) consistently outperforms the other five baseline models in most datasets with the highest F1-score and AUC score. This underscores RF’s attractiveness as a method due to the simplicity of its computations, its efficacy in multi-class problems, and its high performance in terms of F1-score. Additionally, RF’s robustness is enhanced by its ensemble nature.

Secondly, RF also exhibits relatively higher precision and recall across most datasets, with specific exceptions noted in the head and neck dataset for quality-of-life outcomes, and the prostate dataset for bowel pain outcomes. This suggests a lower rate of false positives and false negatives with RF. Conversely, the highest precision rates are recorded by Support Vector Machine (SVM) in the head and neck (quality-of-life) and prostate (Bowel Pain and Sexual Quality) datasets. This indicates that both RF and SVM potentially reduce the number of false positives in such imbalanced multi-class classification scenarios, offering significant promise for managing severe outcomes in cases with fewer patients.

Table 4 reports the comparative analysis of semi-supervised ML, active learning ML, cost-sensitive ML, oversampling ML, and undersampling ML. We found that (1) ML models using oversampling (RF, XGB, GB, SVM, MLP-Bagging) consistently achieve the highest AUC, Precision, Recall, and F1 scores for both Prostate (Bowel Pain) and Breast (Sleeping Discomfort) tasks. Their Precision, Recall, and F1 scores are substantially higher than all other approaches in both categories. (2) SSL-RF and SSL-XGB achieve very competitive AUC scores (0.901, 0.899 for Prostate; 0.848, 0.820 for Breast), often matching or coming close to the best Oversampling results. However, its Precision, Recall, and F1 scores, while decent, are generally lower than Oversampling. (3) For the Prostate task, cost-sensitive models (CSL-SVM, CSL-GB, CSL-LR, CSL-RF, CSL-XGB) achieve the highest Recall scores (0.660–0.700), significantly outperforming other non-oversampling methods. CSL-SVM also achieves the highest F1 score (0.640) for Prostate among non-oversampling methods. (4) Active Learning models (AL-RF, AL-XGB, AL-GB, AL-SVM) generally achieve solid but not leading AUC scores (mostly 0.815–0.892). Their Precision, Recall, and F1 scores are typically lower than both Oversampling and Cost-Sensitive approaches, placing them in the middle of the pack.

For the running time comparison in Table 5, we found that the SVM algorithm was the most efficient, achieving the lowest computational cost among the tested models across three distinct cancer types. This efficiency is followed by LR, RF, MLP-Bagging, XGB, and B, in order. The relatively higher computational times for RF, MLP-Bagging, XGB, and GB can primarily be attributed to their use of grid search strategies for hyper-parameter optimization, which involves exhaustively searching through every possible combination of hyper-parameter values to find the best settings. This thorough search contributes to their increased running times compared to SVM and LR.

We also compared the model complexity of six ML classifiers in the last row of Table 5, where *n* represents the number of instances, *d* denotes the number of features, *k* is the number of trees, *t* is the maximal number of iterations, *L* is the number of hidden layers, and *E* is the number of component classifiers. We observe that the model complexity of LR is linear with *n*, *d*, and *t*, however, SVM is the fastest algorithm as it seeks a convex optimization problem using kernel tricks. The model complexity of RF, XGB, GB, and MLP-Bagging is higher than LR, which consumes more computational costs.

### E. ROC COMPARISON

**FIGURE 3.**
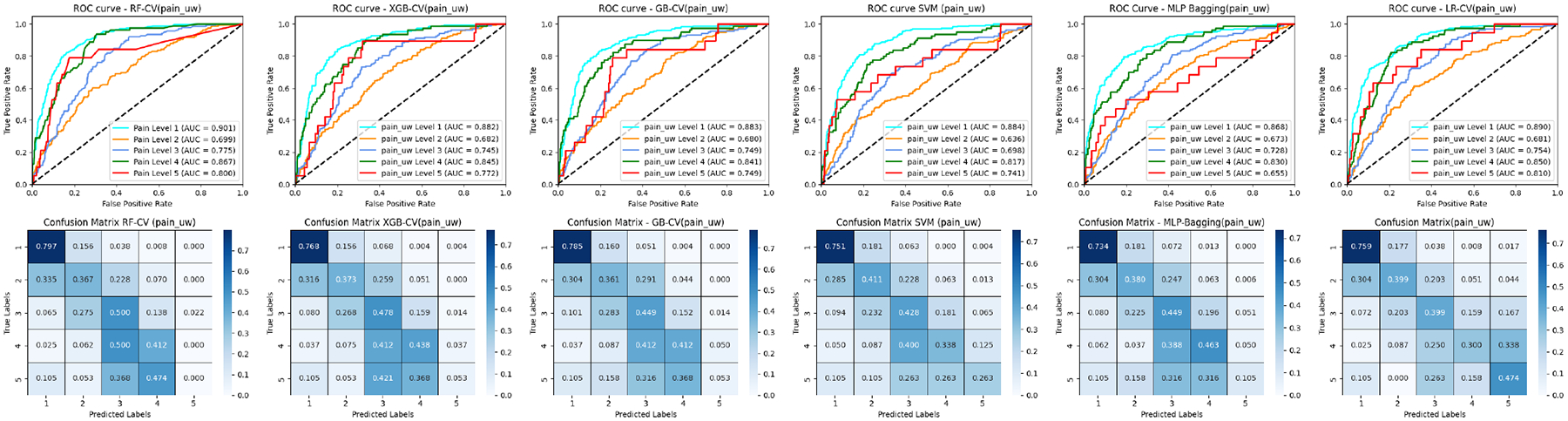
ROC curves and confusion matrices of six ML classifiers for the head and neck cancer patients (pain levels 1–5).

Figures 3, 5, and 7 (upper panel) display the ROC curves for each subclass within three distinct cancer datasets. Our analysis revealed that most methods consistently achieve relatively high AUC scores for pain levels 1 and 4. Notably, RF secures high AUC scores for pain levels 2 and 3, while LR attains the highest AUC scores for pain level 5 when compared to the other five baseline methods. Similar patterns are observed in prostate and breast cancer datasets for pain and sleep discomfort outcomes, respectively. These findings suggest that RF is particularly effective for classifying the majority classes, whereas LR excels in minority classification.

Additionally, the results depicted in Figures 7 and 9 include comparisons of the ROC curves and confusion matrices both with and without oversampling. These comparisons underscore the effectiveness of deep learning-based methods, such as MLP-Bagging, in managing multi-class classification tasks with skewed data distributions. After implementing oversampling to balance the training set, some traditional methods like LR, SVM, and GB demonstrated enhanced performance relative to the deep learning-based approach, as evidenced by the results presented in these figures.

### F. CONFUSION MATRIX COMPARISON

Figures 3, 5, and 7 (lower Panel) display the confusion matrices for each subclass across three cancer datasets. Our analysis shows that nearly all methods attain relatively high classification accuracy for pain levels 1 and 4. Notably, RF demonstrates high accuracy for pain levels 2 and 3, while LR achieves the highest classification accuracy for pain level 5 compared to the other five baseline methods. For the patients with similar pain levels, almost all classifiers suffer a low classification accuracy while a high over or underrate classification (for example pain levels 3 and 4) as demonstrated in the matrix with adjacent positions. This indicates that incorrect evaluation of similar pain levels from patients may increase the difficulty for the classifiers to make the correct prediction. Similar trends are noted in the prostate and breast cancer datasets concerning pain and sleep discomfort outcomes, respectively. These findings reinforce the conclusion that RF is particularly suited for classifying majority classes, whereas LR excels in minority class classification among the baseline methods.

**FIGURE 4.**
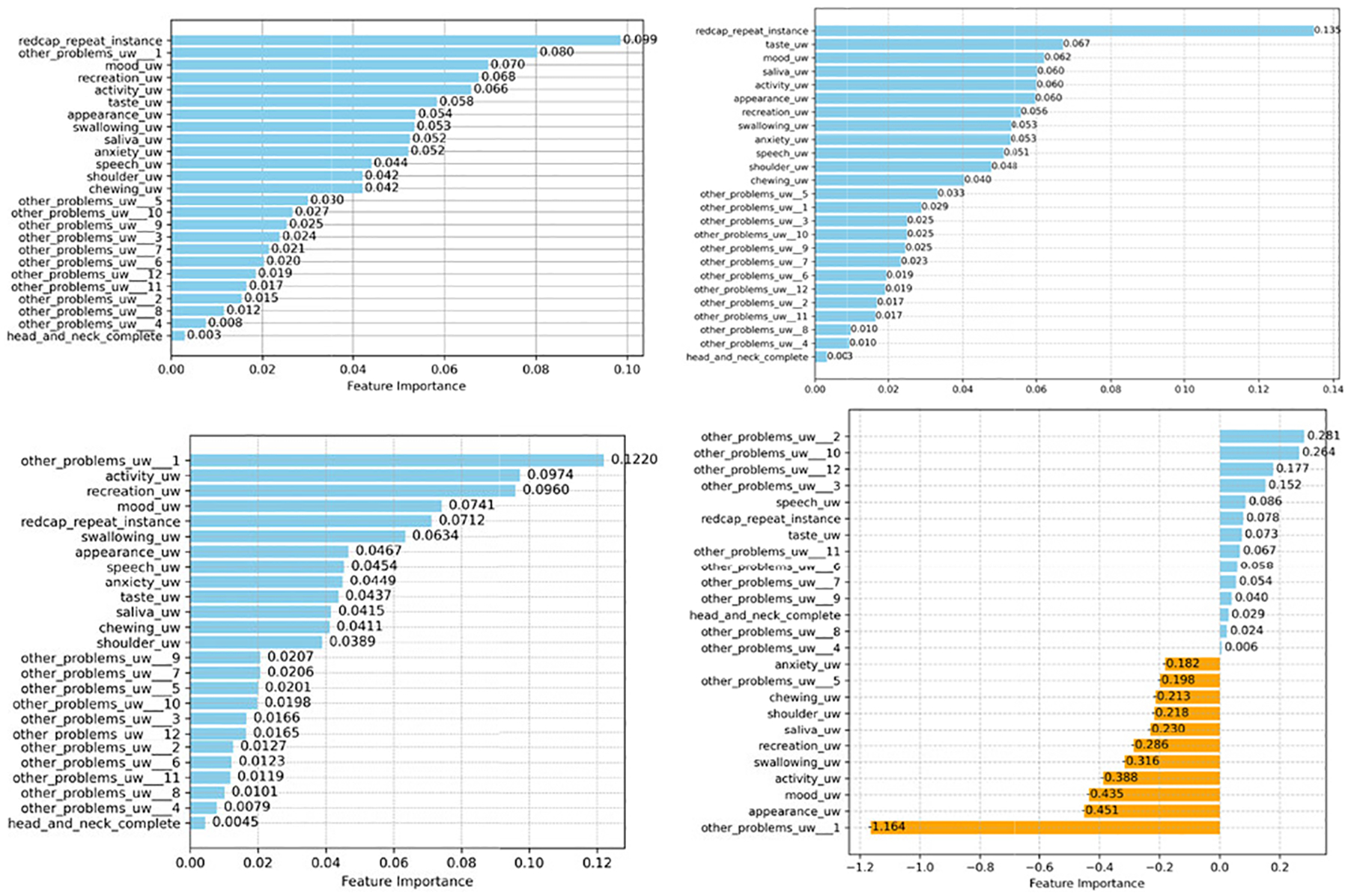
Feature importance scores of four ML classifiers on the head and neck cancer PRO (Top left RF; Top right XGB; Bottom left GB; and Bottom right LR). For tree-based models (RF, XGB, GB), each blue bars indicate the normalized feature importance (all coefficients sum to 1). For LR, each bar shows the standardized coefficient of each PRO features. Blue indicates the positive coefficient and orange shows negative coefficient.

**FIGURE 5.**
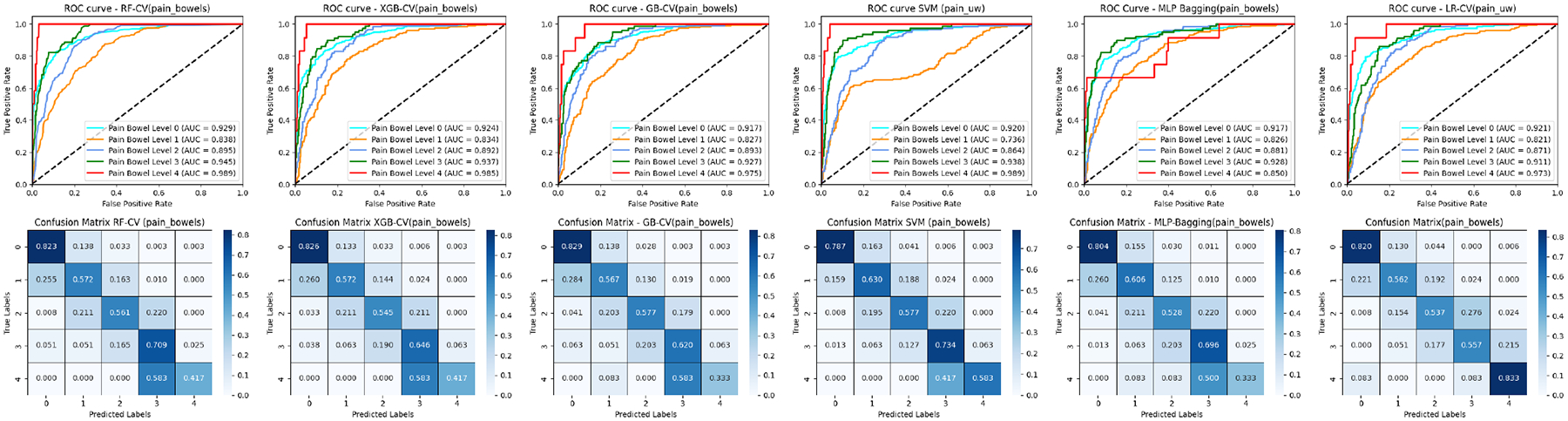
ROC curves and confusion matrices of six ML classifiers for the prostate cancer patients (bowel pain levels 0–4).

**FIGURE 6.**
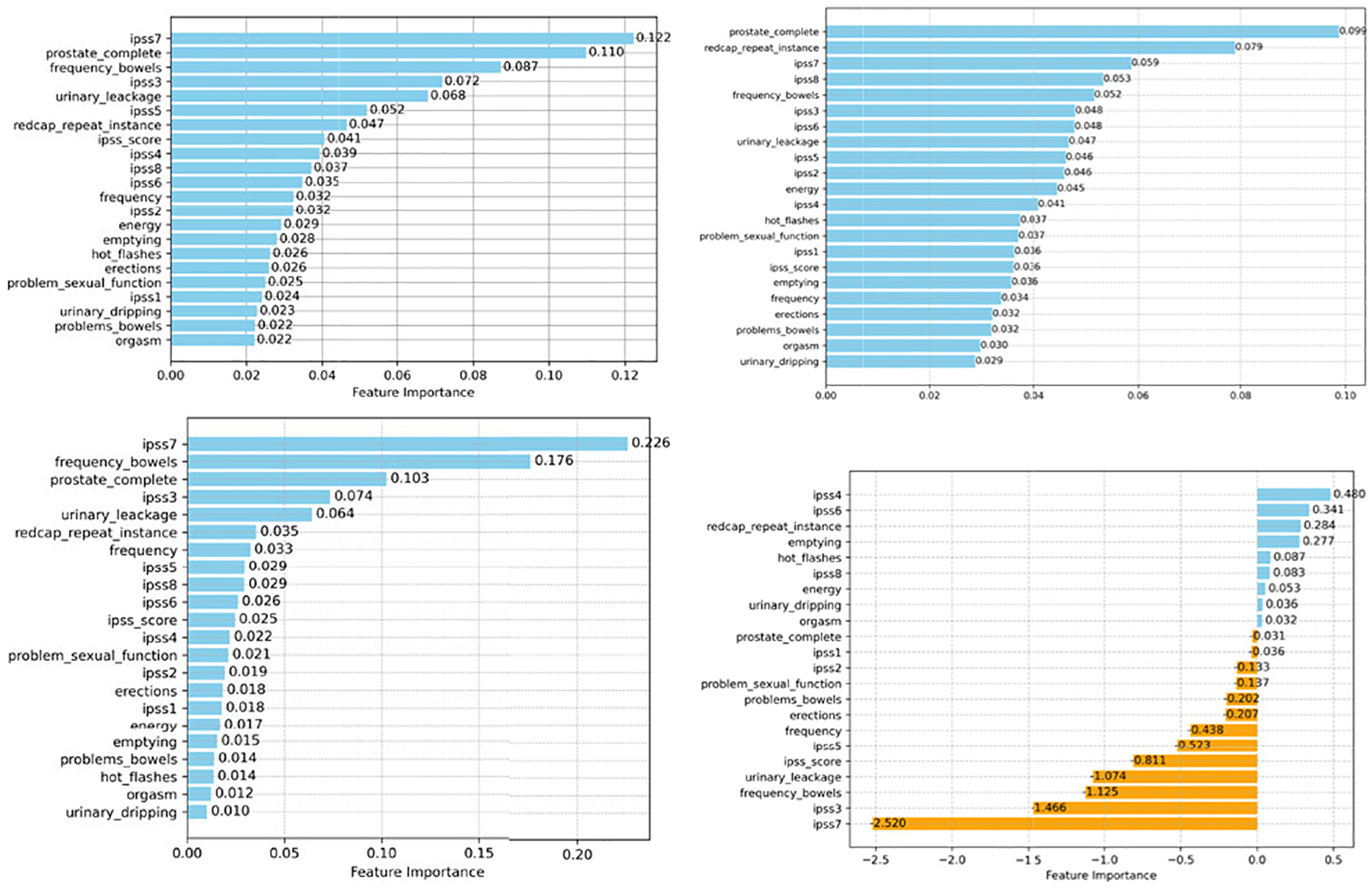
Feature importance scores of four ML classifiers on the prostate cancer (Top left RF; Top right XGB; Bottom left GB; and Bottom right LR).

Additionally, we examined the importance scores for RF, XGB, GB, and LR in Figures 4, 6, and 8. We found that the feature “redcap-repeat-instance” is among the most significant attributes for RF and XGB in enhancing classification performance. The ranking of features along with their respective importance scores or coefficients offers valuable insights into the functioning and utility of these ML classifiers, providing a clearer understanding of their predictive capabilities within the PRO datasets.

### G. SEMI-SUPERVISED ML WITH OVERSAMPLING FOR PRO

**FIGURE 7.**
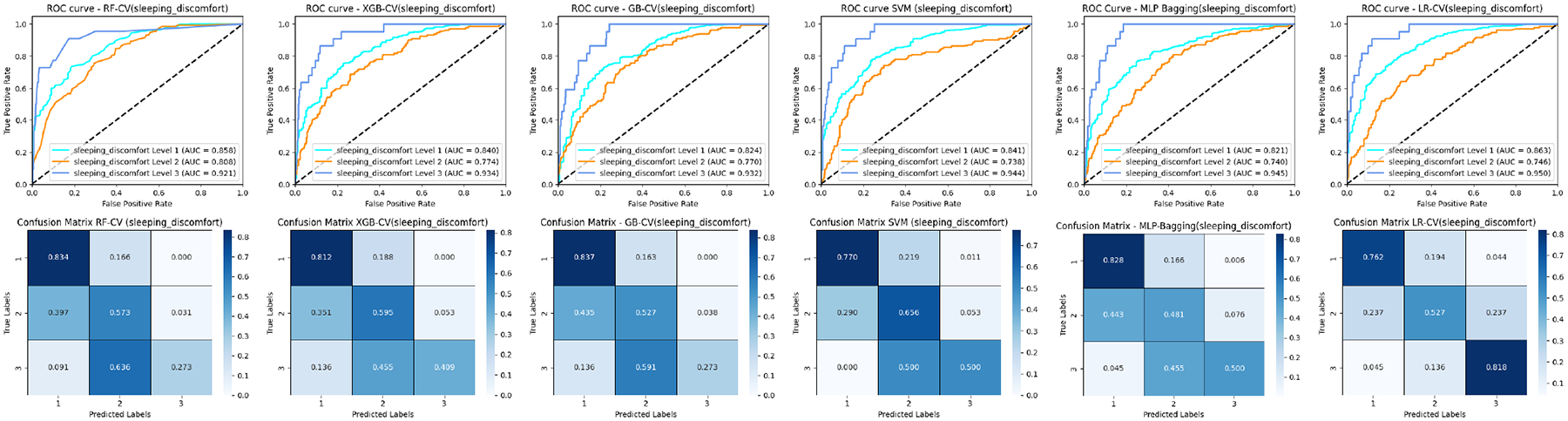
ROC curves and confusion matrices of six ML classifiers for the breast cancer patients (sleeping discomfort levels 1–3).

**FIGURE 8.**
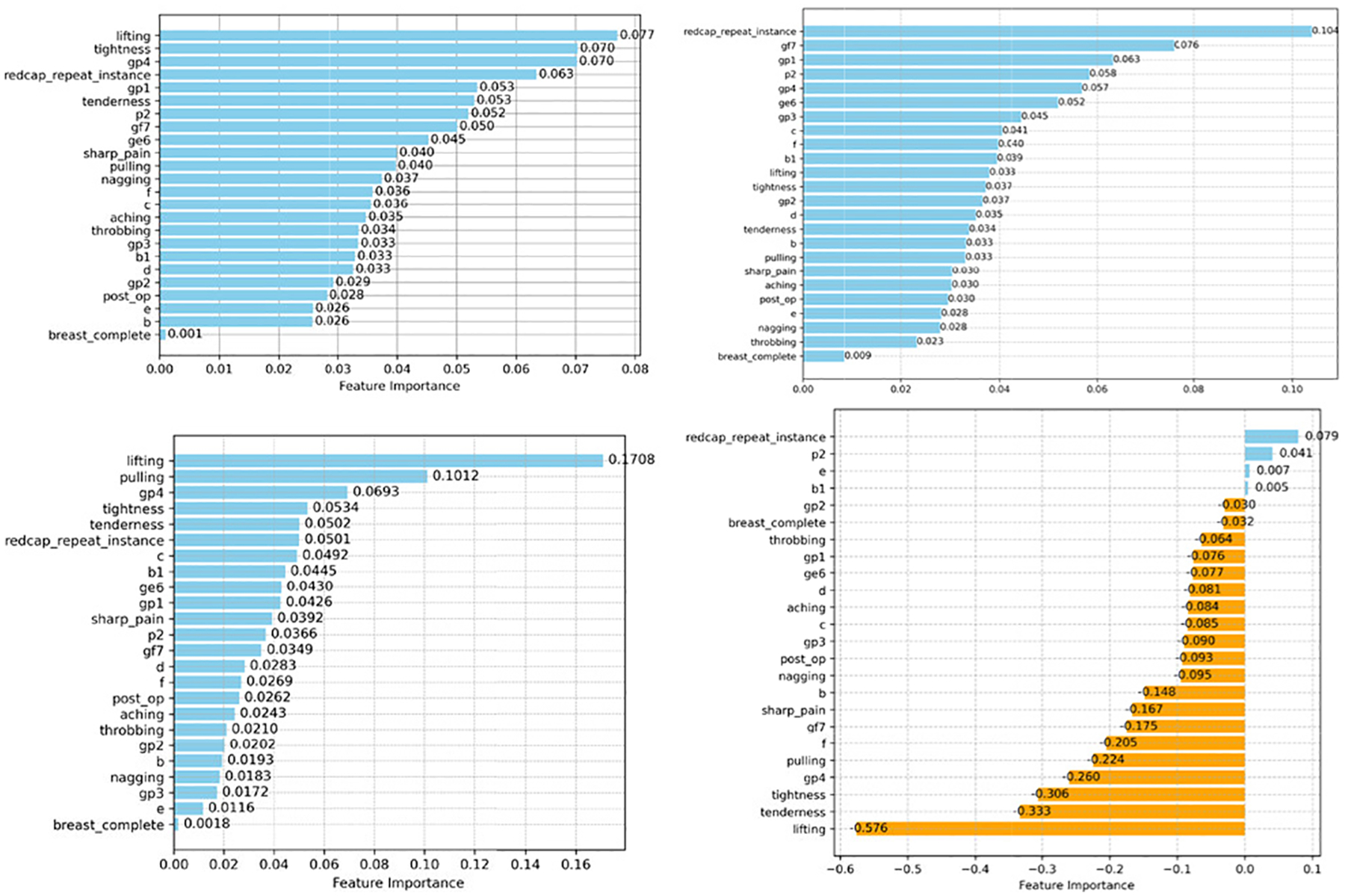
Feature importance scores of four ML classifiers on the breast cancer (Top left RF; Top right XGB; Bottom left GB; and Bottom right LR).

**FIGURE 9.**
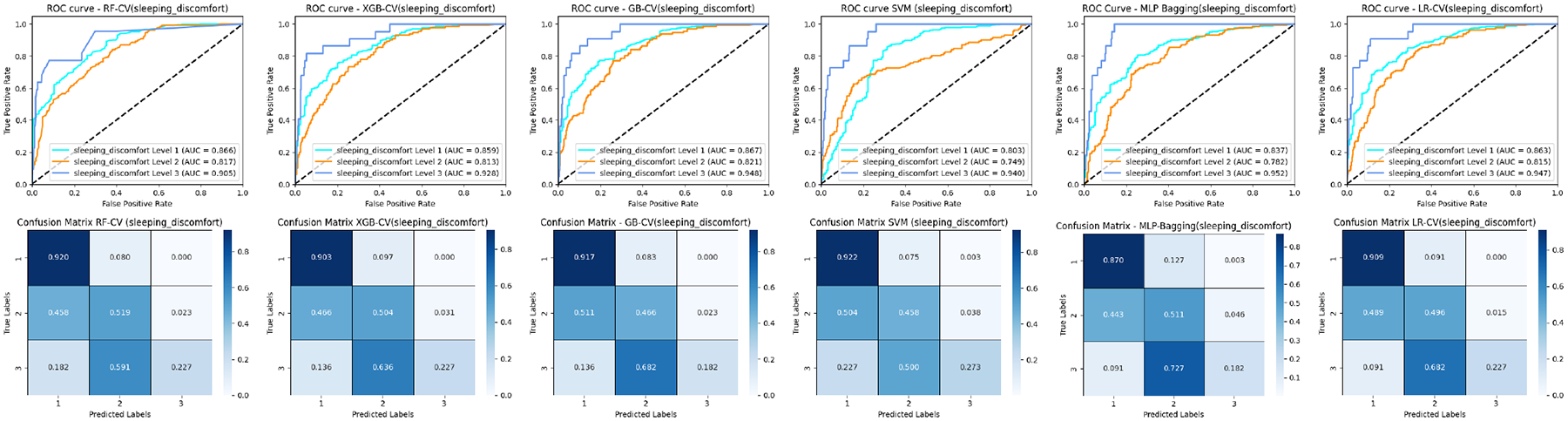
ROC curves and confusion matrices of six ML classifiers without oversampling for the breast cancer patients (sleeping discomfort levels 1–3).

**FIGURE 10.**
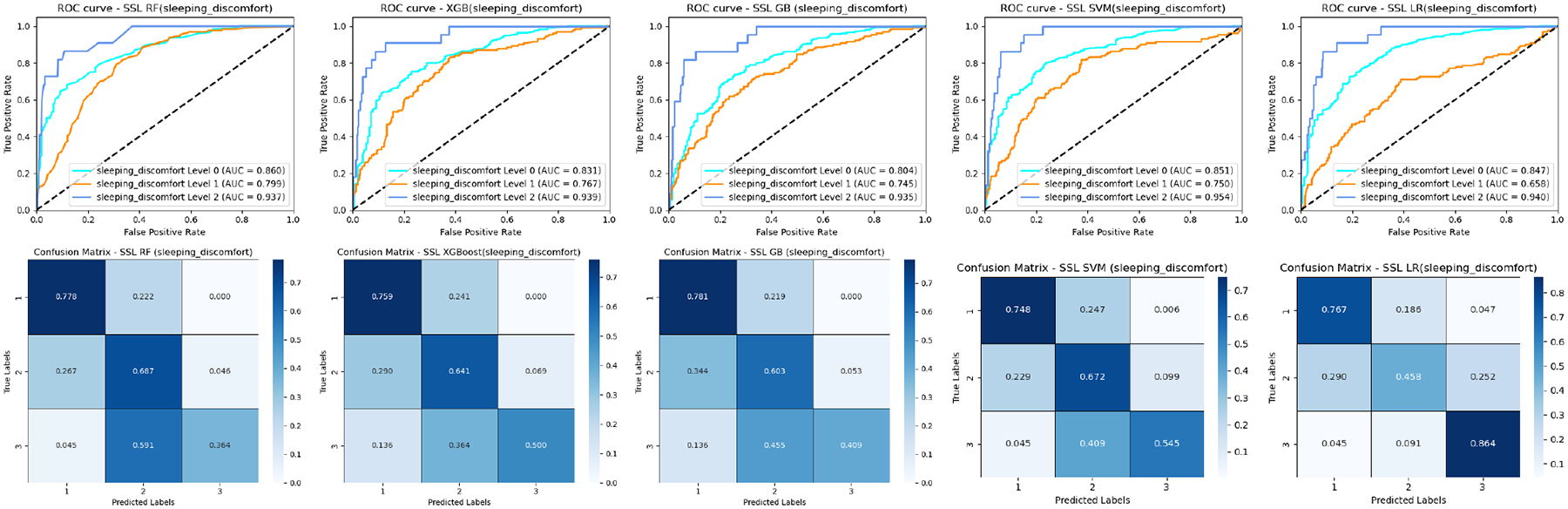
ROC curves and confusion matrices of five semi-supervised ML classifiers for the breast cancer patients (sleeping discomfort levels 1–3).

Using a similar semi-supervised learning strategy in [54], we investigate the impact of partially labeling incomplete PRO responses to demonstrate feasibility and discuss how semi-supervised ML could prioritize minority-class samples for annotation, reducing labeling burdens while mitigating imbalance. Figure 10 shows the results on breast cancer with different sleeping discomforts. For SSL-RF, SSL-XGB, SSL-GB, SSL SVM, we found that the prediction accuracy of patients with sleeping discomfort levels 2 and 3 has been significantly increased while the prediction accuracy of patients with sleeping discomfort level 1 has been slightly reduced, especially comparing SSL-LR (Figure 10) and LR (Figure 7). This indicates that the semi-supervised learning strategy may alleviate the class imbalance issue by reducing the overfitting to the majority class (e.g., sleeping discomfort level 1), and boost recall on the underrepresented class (e.g., sleeping discomfort levels 2 and 3).

### H. ACTIVE MACHINE LEARNING WITH OVERSAMPLING FOR PRO

To enhance data efficiency in supervised learning, we implemented an active leaning strategy on the Query-By-Committee (QBC) framework [57] using six machine learning classifiers. In this setup, a committee of diverse learners was trained on bootstrapped subsets of an initially small labeled dataset. This learning process iteratively queried the most informative instances from a large unlabeled pool, guided by the vote entropy criterion, which measures disagreement among committee members. This approach enables the model to focus annotation efforts on the samples that are expected to provide the most utility for learning. The initial training subset comprised 10% of the available training data. From this point, the model performed 10 rounds of querying, selecting 50 uncertain instances per round. Each round involved: (1) Estimating label disagreement using vote entropy. (2) Querying the most uncertain samples. (3) Simulating oracle labeling using the ground truth. (4) Incrementally updating all learners in the committee. The final committee was evaluated on a separate test set.

**FIGURE 11.**
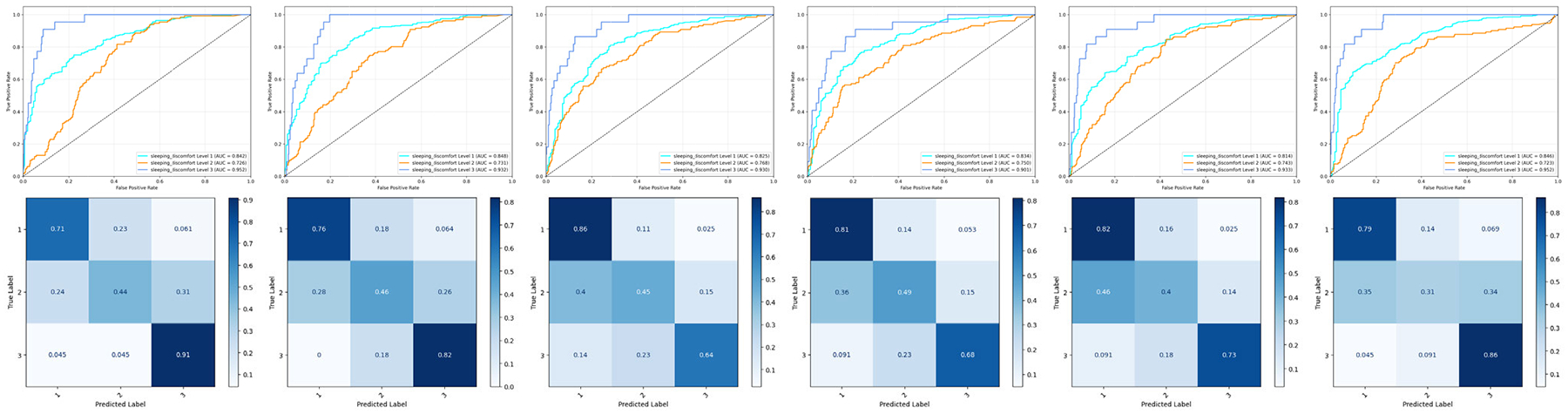
ROC curves and confusion matrices of six ML classifiers with QBC (Top Row AL-RF; AL-XGB; AL-GB; Bottom Row AL-SVM; AL-MLP-Bagging; AL-LR) for the breast cancer patients (sleeping discomfort levels 1–3).

Figure 11 shows the results of breast cancer with sleeping discomforts. We observed that the predicted accuracy of patients with sleep discomfort level 3 has increased significantly while the trade-off is the performance to the predicted accuracy of patients with sleep discomfort level 2, especially when comparing AL-RF and AL-XGB (Figure 11) and RF and XGB (Figure 7), respectively. The predicted accuracy for patients with sleep discomfort level 3 by AL-RF and AL-XGB was increased from 0.273 and 0.409 to 0.91 and 0.82. These results confirm that QBC not only increases minority class recognition but also reduces prediction bias toward dominant classes.

### I. COST-SENSITIVE MACHINE LEARNING FOR PRO ANALYSIS

**FIGURE 12.**
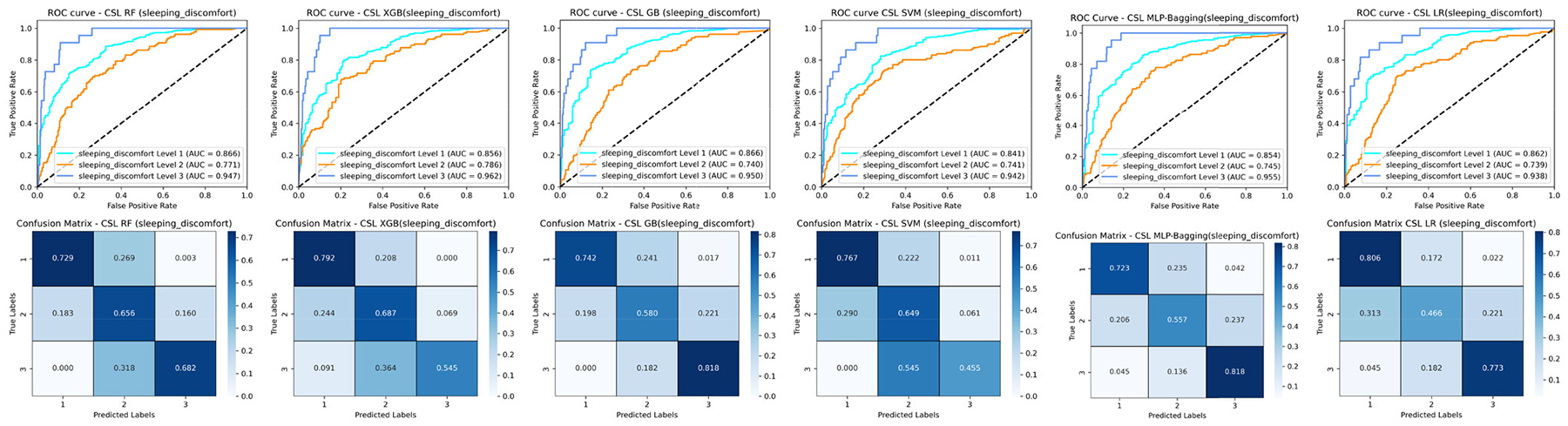
ROC curves and confusion matrices of six ML classifiers with cost-sensitive learning for the breast cancer patients (sleeping discomfort levels 1–3).

Cost-sensitive learning [56] addresses class imbalance by incorporating misclassification costs directly into the training process, enhancing model sensitivity to minority classes. For GB or XGB, this involves adjusting class weights (e.g., scale_pos_weight) or modifying the loss function to penalize minority class errors more heavily, respectively. In LR or SVM, class-specific weights (e.g., class_weight in scikit-learn) are applied to the logistic loss or margin penalties, respectively, to prioritize minority samples. RF and tree-based methods integrate cost sensitivity by biasing splits using metrics like weighted Gini impurity or entropy. MLP can employ weighted loss functions during backpropagation, dynamically scaling errors for minority instances. By embedding cost-aware adjustments into these algorithms—whether via reweighted loss functions, split criteria, or hyperparameter tuning—models like GB, LR, MLP, RF, SVM, and XGB achieve improved generalization on imbalanced PRO datasets, balancing precision-recall trade-offs and boosting metrics including ROC curve and confusion matrix (Figure 12) while mitigating bias toward majority classes. We observe that GB and MLP-Bagging with a cost-sensitive learning strategy will enhance their predicted performance for breast cancer patients with severe sleep discomfort, they achieved highest predicted accuracy for sleep discomfort level 3. RF (0.573), XGB (0.595) and MLP-Bagging (0.481) with cost-sensitive learning perform better on classify the sleep discomfort level 2 compared to RF (0.656), XGB (0.687) and MLP-Bagging (0.557) (Figure 7). However, these four approaches (RF, XGB, GB, and MLP-Bagging) with a cost-sensitive learning strategy will slightly reduce prediction performance for breast cancer patients with sleep discomfort levels 1 compared to supervised learning, which is similar to semi-supervised learning with oversampling.

The possible reasons for degradation can be summarized as below: (1) CSL works by increasing the cost of misclassifying the minority class. If the cost ratio is set too aggressively, the model becomes hyper-focused on correctly classifying the minority class, potentially by learning noise or rare, ungeneralizable patterns in the minority class. (2) The performance of CSL is highly sensitive to the specific cost values assigned. Using arbitrary, non-data-driven costs, or costs not reflecting the true operational imbalance or misclassification consequences, leads to poor generalization. (3) For the tree-based methods (RF, XGB, GB), cost integration might primarily affect the splitting criteria or instance weighting. If the cost structure doesn’t align well with the impurity measure (e.g., Gini, Entropy) or boosting objectives, it can distort tree building, leading to suboptimal partitions that reduce overall AUC. The corresponding mitigation strategies for CSL can be resolved by refining cost matrix definition, combining CSL with resampling, or threshold moving with post-calibration. For example, train a model (even cost-insensitive) with good discriminative power (AUC), calibrate its probabilities (e.g., Platt Scaling, Isotonic Regression [65]), and then adjust the decision threshold to favor the minority class after training. Rigorously tune core hyper-parameters (e.g., tree depth, learning rate, regularization for XGB/GB; network architecture, dropout for MLP) in conjunction with the cost matrix.

### J. PARAMETER SENSITIVITY STUDIES

**FIGURE 13.**
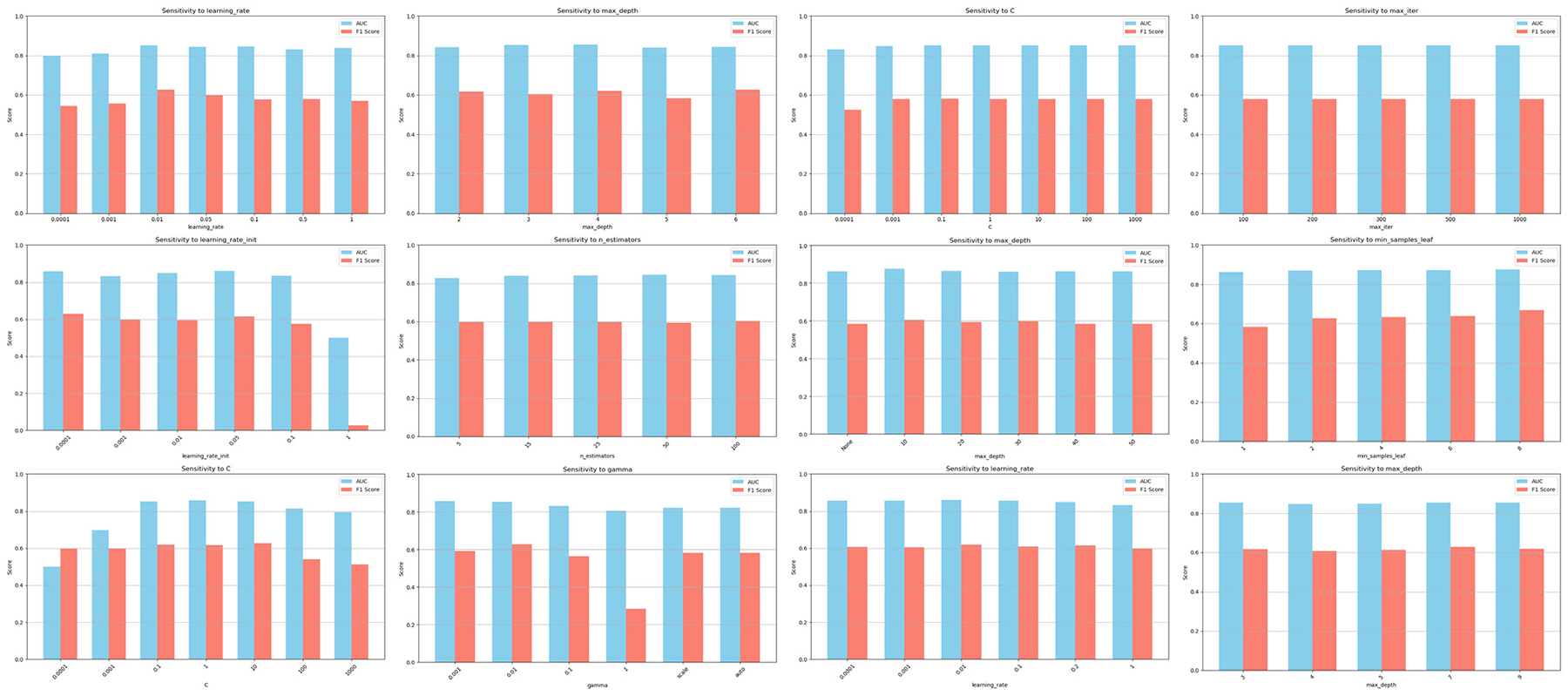
Parameter sensitivity analysis of GB (Row 1, Columns 1–2), LR (Row 1 Columns 3–4), MLP-Bagging (Row 2, Columns 1–2), RF (Row 2, Columns 3–4), SVM (Row 3, Columns 1–2), and XGBoost (Row 3, Columns 3–4) on the PRO reports with breast cancer.

For each algorithm, we have specified the hyper-parameters optimized (e.g., regularization strength and solver for LR; hidden layers, activation functions, and dropout rates for MLP; tree depth, ensemble size, and splitting criteria for RF/XGBoost/GB; kernel type, penalty, and gamma for SVM) and the tuning strategy employed (e.g., Bayesian optimization for XGBoost, grid search for SVM). We have clarified that optimization was guided by stratified 5-fold cross-validation on the training set only, prioritizing class-imbalance-aware metrics (e.g., F1-score, AUC-score). For reproducibility, we have detailed search spaces (e.g., XGBoost’s learning rate: [0.0001, 0.001, 0.01, 0.1, 0.2, 1]; RF’s max depth: [none, 10, 20, 30, 40, 50]), tools (e.g., scikit-learn’s GridSearchCV, Optuna), and validation safeguards (e.g., early stopping for MLP/XGBoost to prevent overfitting) in Figure 13. We found that GB is sensitive to learning rate (Small or Large learning rate will cause AUC/F1 dropping) while is relatively robust to max depth. LR is robust to *C* and max number of iterations. MLP-Bagging is sensitive to learning rate while is relatively robust to the number of estimators. RF and XGBoost are insensitive to the parameter settings. SVM is sensitive to parameters *C* and *γ* of the RBF kernel, where the best parameter combination is *C* = 1 and *γ* = 0.01. Additionally, we have discussed sensitivity analyses (e.g., performance variations across hyperparameter ranges) and justified selections based on robustness to imbalance (e.g., XGBoost’s learning rate and max depth adjustments).

## V. CONCLUSION

This study demonstrates the efficacy of applying diverse ML techniques and addressing class imbalance through strategic oversampling to enhance predictive accuracy for clinical outcomes (e.g., patient health trajectories) in cancer patients undergoing radiation therapy. By tailoring classifiers to account for cancer type variability and symptom severity, we developed a suite of robust ML models for PRO analysis. The RF and XGB models not only improved PRO forecasting but also supported clinical decision-making through their inherent interpretability. LR demonstrated strong performance in classifying patients with severe health deterioration, suggesting its utility for high-risk cohorts. Our hybrid imputation and oversampling framework outperformed non-oversampling approaches, offering a potential benchmark for future predictive analytics in PRO research. We also investigated semi-supervised learning, cost-sensitive learning, and active learning strategies to further improve the prediction performance, especially for patients with severe health conditions from the PRO reports during radiation therapy.

Future work will explore anomaly detection in PRO data to identify high-risk cancer patients with severe health outcomes during radiation therapy. Additionally, we plan to leverage semi-supervised anomaly detection methods to predict critical health deterioration in scenarios with limited labeled clinical data, addressing a key challenge in real-world oncology practice.

## Figures and Tables

**TABLE 1.** Summary of the PRO datasets utilized in the experiments.

Dataset	#Attributes	#Training Instances	#Training Instances (Oversampling)	#Testing Instances	#Classes
Head&Neck (pain)	25	2,525 (939:657:554:304:71)	4,695 (939:939:939:939:939)	632 (237:158:138:80:19)	5
Prostate (bowel pain)	21	3,139 (1,477:767:491:339:65)	7,385 (1,477:1,477:1,477:1,477:1,477)	784 (362:208:123:79:12)	5
Breast (sleeping)	24	2,054 (1,430:555:69)	4,290 (1,430:1,430:1,430)	514 (361:131:22)	3

**TABLE 2.** The Parameters of Traditional Machine Learning Algorithms and Their Parameter Value Ranges.

Method	Parameter	Value Range
RF	N estimators	[10, 50, 100, 200]
	Max features	[sqrt, log2]
	Max depth	[None, 10, 20, 30,40,50]
	Min samples split	[2, 5, 10]
	Max samples leaf	[1, 2, 4]
XGB	N estimators	[50, 100, 200]
	Learning rate	[0.01, 0.1, 0.2]
	Max depth	[3, 4, 5]
	Min child weight	[1, 2, 3]
	Gamma	[0, 0.1, 0.2]
	Subsample	[0.8, 0.9, 1.0]
	Colsample bytree	[0.8, 0.9, 1.0]
GB	N estimators	[50, 100, 200]
	Learning rate	[0.01, 0.1, 0.2]
	Max depth	[3, 4, 5]
	Min samples split	[2, 4]
	Max samples leaf	[1, 2]
	Max features	[sqrt, log2, None]
SVM	Kernel	rbf
	C	10
	Decision function shape	ovr
	Gamma	0.01
MLP-Bagging	Max iteration	1000
	Initial learning rate	0.001
	Solver	adam
	Estimator	base_mlp
	N estimators	10
LR	C	[0.1, 1, 10, 100, 1000]
	Solver	[newton-cg, lbfgs, sag, saga]
	Max iterations	[100, 200, 300, 500, 1000]

**TABLE 3.** Precision, Recall, F1-, and AUC-score comparison of six ML classifiers on three cancer PRO datasets. The best results are highlighted in bold.

Method	Metric	H&N (pain)	H&N (QoL)	Pro (bowel pain)	Pro (depression)	Pro (sexual)	Breast (muscle)	Breast (sleeping)
RF	Precision	0.533	0.498	0.702	**0.454**	0.661	**0.787**	0.750
	Recall	**0.553**	0.494	0.696	**0.460**	**0.672**	**0.785**	**0.740**
	F1-score	**0.541**	**0.494**	0.697	**0.455**	**0.662**	**0.784**	0.744
	AUC	**0.815**	0.776	**0.902**	**0.761**	**0.883**	**0.845**	**0.848**
XGB	Precision	0.527	0.497	0.691	0.419	0.660	0.756	**0.755**
	Recall	0.542	0.488	0.691	0.417	0.665	0.759	0.739
	F1-score	0.536	0.488	0.692	0.416	0.660	0.760	**0.741**
	AUC	0.794	**0.785**	0.898	0.734	0.878	0.829	0.827
GB	Precision	0.524	0.491	0.688	0.406	0.639	0.741	0.731
	Recall	0.535	**0.496**	0.693	0.411	0.646	0.748	0.737
	F1-score	0.526	0.492	0.688	0.407	0.641	0.746	0.735
	AUC	0.794	0.761	0.891	0.719	0.867	0.806	0.815
SVM	Precision	0.526	**0.518**	**0.722**	0.420	**0.671**	0.744	0.764
	Recall	0.521	0.485	**0.705**	0.422	0.637	0.746	0.730
	F1-score	0.519	0.492	**0.711**	0.419	0.647	0.756	0.740
	AUC	0.769	0.783	0.865	0.726	0.850	0.802	0.819
MLP-Bagging	Precision	0.522	0.483	0.696	0.429	0.649	0.750	0.730
	Recall	0.525	0.472	0.690	0.427	0.649	0.741	0.727
	F1-score	0.522	0.476	0.694	0.427	0.649	0.750	0.726
	AUC	0.778	0.758	0.886	0.720	0.863	0.813	0.805
LR	Precision	**0.544**	0.469	0.698	0.339	0.649	0.757	0.769
	Recall	0.524	0.418	0.681	0.343	0.631	0.722	0.704
	F1-score	0.528	0.425	0.686	0.327	0.630	0.739	0.723
	AUC	0.801	0.719	0.757	0.683	0.780	0.719	0.837

**TABLE 4.** Precision, Recall, F1-score, and weighted AUC-score comparison of six ML classifiers with different strategies on prostate and breast cancer PRO datasets. The best results on each dataset within same learning strategy are highlighted in bold.

Category	Model	Prostate (Bowel Pain)				Breast (Sleeping Discomfort)			
		AUC	Precision	Recall	F1	AUC	Precision	Recall	F1
Semi-supervised ML	SSL-RF	**0.901**	**0.597**	0.599	0.594	**0.848**	**0.650**	0.608	0.616
	SSL-XGB	0.899	0.572	0.581	0.575	0.820	0.605	0.592	0.591
	SSL-GB	0.892	0.554	0.563	0.557	0.794	0.629	0.598	0.606
	SSL-SVM	0.888	0.587	**0.610**	**0.597**	0.830	0.605	0.655	**0.620**
	SSL-LR	0.850	0.481	0.584	0.493	0.803	0.539	**0.696**	0.566
Active ML	AL-RF	0.891	**0.568**	0.611	**0.582**	0.817	0.513	**0.688**	0.532
	AL-XGB	**0.892**	0.554	0.630	0.560	**0.822**	0.529	0.678	0.549
	AL-GB	0.876	0.553	0.570	0.557	0.815	**0.579**	0.649	**0.595**
	AL-SVM	0.883	0.556	0.591	0.563	0.815	0.557	0.660	0.580
	AL-MLP-Bagging	0.860	0.509	0.544	0.503	0.801	0.554	0.650	0.582
	AL-LR	0.885	0.553	**0.636**	0.561	0.819	0.505	0.656	0.512
Cost-sensitive ML	CSL-RF	0.895	0.574	0.638	0.596	**0.845**	0.593	**0.690**	0.620
	CSL-XGB	**0.902**	0.574	0.638	0.586	0.843	**0.660**	0.677	**0.663**
	CSL-GB	0.899	0.586	0.672	0.610	0.777	0.523	0.650	0.550
	CSL-SVM	0.868	**0.610**	**0.700**	**0.640**	0.800	0.563	0.680	0.597
	CSL-MLP-Bagging	0.876	0.542	0.622	0.566	0.780	0.527	0.643	0.553
	CSL-LR	0.888	0.562	0.660	0.584	0.806	0.527	0.667	0.553
Oversampling ML	RF	**0.902**	0.702	0.696	0.697	**0.848**	0.750	**0.740**	**0.744**
	XGB	0.898	0.691	0.691	0.692	0.827	0.755	0.739	0.741
	GB	0.891	0.688	0.693	0.688	0.815	0.731	0.737	0.735
	SVM	0.865	**0.722**	**0.705**	**0.711**	0.819	0.764	0.730	0.740
	MLP-Bagging	0.886	0.696	0.690	0.694	0.805	0.730	0.727	0.726
	LR	0.757	0.698	0.681	0.686	0.837	**0.769**	0.704	0.723
Downsampling ML	RF	0.869	0.524	**0.608**	**0.550**	**0.823**	**0.573**	0.680	**0.603**
	XGB	**0.875**	**0.540**	0.598	0.530	0.818	0.523	**0.693**	0.543
	GB	0.846	0.490	0.560	0.508	0.777	0.523	0.650	0.550
	SVM	0.838	0.508	0.582	0.528	0.800	0.563	0.680	0.597
	MLP-Bagging	0.851	0.484	0.552	0.504	0.780	0.527	0.643	0.553
	LR	0.856	0.494	0.586	0.514	0.806	0.527	0.667	0.553
Raw ML	RF	0.903	0.646	0.582	0.598	0.855	0.693	**0.557**	**0.590**
	XGB	**0.907**	0.596	0.570	0.576	0.851	0.657	0.543	0.577
	GB	0.905	0.598	0.554	0.566	**0.859**	0.650	0.517	0.547
	SVM	0.875	0.640	0.592	0.610	0.795	0.643	0.550	0.580
	MLP-Bagging	0.891	**0.660**	**0.602**	**0.618**	0.828	0.573	0.520	0.537
	LR	0.740	0.544	0.496	0.502	0.855	**0.707**	0.547	0.580

**TABLE 5.** Training Time (seconds) and Model Complexity Comparison of six machine learning classifiers on three cancer PRO datasets.

Method	RF	XGB	GB	SVM	MLP-Bagging	LR
**Head&Neck (pain)**	353.18	3815.68	1972.74	0.35	825.15	11.49
**Prostate (bowel pain)**	594.53	2167.71	3513.67	0.54	1039.73	26.71
**Breast (sleeping)**	301.17	909.25	1071.57	0.19	691.33	9.87
**Model Complexity**	𝒪(k⋅nlogn⋅d)	𝒪(k⋅nlogn⋅d)	𝒪(k⋅nlogn⋅d)	$𝒪\left(n^{2}\cdot d\right)$	$𝒪\left(E\cdot L\cdot n\cdot d^{2}\cdot t\right)$	𝒪(n⋅d⋅t)
